# Current Hospital Topics

**Published:** 1907-05-18

**Authors:** 


### CURRENT HOSPITAL TOPICS.

### Bristol General Hospital.

Amongst older hospitals in the provinces, few present more interesting and attractive features to the observant than this institution. The system of electing the medical officers by a committee of governors, elected annually, is modern and up-to-date. There is an active Ladies' Committee, and the Medical Departments of the Hospital include one each for the Throat and Nose, for the Skin, the Eye, the Ear, Diseases of Women, Electric Light Treatment, Bacteriology and Pathology. The report is attractively illustrated, well printed and full of interest. We are surprised, however, to notice that, the accounts are not kept upon the Uniform System, which places the institution under a disadvantage, as its work cannot be readily compared with that of similar institutions, which must prove a drawback to the Hospital in many ways. We should recommend the appointment of an economy committee, which might take up the whole question of accounts, the purchase of stores, dieting, and a large number of other items, with results which we think might possibly surprise some of its most zealous supporters. We are glad to see in this report the portraits of the President and the Treasurer, two Bristol worthies, in the persons of Mr. William Proctor Baker and Mr. Joseph Storrs Fry. The report reflects great credit upon the Committee and its Secretary. The Bristol General Hospital too, has been unusually fortunate for many years in the *personnel* of its Nursing Staff, the head of which has usually been a lady of marked administrative ability and acumen. The present Matron, Miss Sophy Morris, is a worthy successor to several eminent predecessors.

### Westminster Hospital.

This Hospital has issued its 189th report, a fact which must commend the institution to the sympathy of all Londoners. It is often felt, that, a hospital, situated in the heart of a large town, cannot be an ideal residence for sick people, hygienically. At the Westminster Hospital in 1906, however, although upwards of 200 more patients were admitted to in-patient treatment, the average number of beds occupied for the whole year was 8 less than in 1905, a fact which we attribute to the circumstance that, the mean residence of each in-patient was under 25 days, a reduction of one and a half days per each patient compared with the previous year. The Committee attributes the slight decrease, about one-twenty-fifth, in the number of out-patients, to the prolonged summer weather, but we hope it may be due in a large measure to the efficiency of the work done by the almoner. The Committee properly express much regret at the retirement of Miss Nussey, who has filled the office of Lady Almoner for seven years with marked efficiency. Her successor is Mrs. Crace, who it is hoped will show equal skill and tact in dealing with the patients, and equal helpfulness in obtaining for them convalescent treatment and surgical appliances, where necessary. That the Hospital has up-to-date Departments, is indicated by the circumstance that, upwards of 300 more patients were treated in the Electrical Branch last year, than in 1904. A point is made of the fact that some forms of diseases of the skin which entailed protracted treatment in former years, are now quickly cured by the application of Röntgen Rays. Unfortunately, the ordinary expenditure exceeded the ordinary income by £5,400. The average cost of each occupied bed is stated to have been £92 7s. 3d. in 1906, an increase of about 5s. per bed as compared with the cost in 1905.

### A Much-needed and Growing Hospital.

Wandsworth owes a heavy debt to Canon Erskine Clarke, Chairman of the Bolingbroke Hospital, which he not only founded but has largely supported during many years, in addition to the heavy claims made upon him by parochial duties and the care of several churches, all of which responsibilities he has discharged with exemplary diligence and success. It is one of the most remarkable features of the Church of England from a layman's point of view that, Canon Erskine Clarke should not have been promoted to high office in the Church, for there are few better men of business among the clergy, few more generous spirits, and few whose administrative gifts can compare with his own. The growth of the Bolingbroke Hospital has been steady and increasing. Eight years ago, only 217 accidents and emergency cases were admitted, but in 1906 they amounted to 654. No better evidence could be afforded of the urgent necessity which has grown up for the erection of the new wing which is now approaching completion. Ministering to the needs of a poor population of probably 400,000 people, it is lamentable, though not surprising, to find from the report that, the completion of the new building has been delayed for want of money, for any intelligent and wealthy man of business, who desires to do real good with his money, would find at Wandsworth an opportunity of a very high order. Indeed, the more closely the work is investigated and the better it is understood, the larger is likely to be the contribution of intelligent givers. Wandsworth is a district which has greatly changed of late years, by the influx of an ever-increasing number of poor people, who have changed the whole character of a large portion of the district, which used to contain the residences of many well-to-do and wealthy people. The poorer the district, the greater the need for adequate hospital accommodation, a fact which we commend to the many large-hearted and generous supporters of hospitals, who may be blessed with a superabundance of this world's goods.

### The Annual Report of the Hospital.

We have often felt that the report of many hospitals might be made far more interesting than it is at present. In the older institutions it would very often be an advantage to appoint a small editorial committee including the most literary minds available, to whom the report in its entirety might be referred, with instructions to suggest novelties and improvements in its form and contents. Each hospital is a small republic in itself, and the daily life in the course of each year presents some novel and many amusing features, which should help to illustrate the work, and so to instruct the governors. Where the report has literary merits and attractive features, the custom of sending a copy to each subscriber and governor annually, is, frequently, productive of renewed and increased contributions, and indirectly of securing legacies. Some hospitals which are kept up to date appreciate the points we have urged, and their reports leave little to desire. The Poplar Hospital for Accidents in this respect may be regarded as a pioneer. Each year the report has a new cover, carefully thought out and well designed and attractive. No point that can be made to tell in its favour is omitted, and the type and paper, as well as the contents, exhibit no little ability. We are not sure that we like the departure in this year's report of putting the income and expenditure account at the beginning, though of course finance is the bed-rock upon which every prosperous voluntary hospital must rest. The fifty-first report commences with a paragraph in red ink urging subscribers to come and see the hospital, and referring them to a plan on the cover, which shows them how to get there. This is a good idea and might be generally adopted. Then there is an appeal for a new Convalescent Home, which is tersely and forcibly put. It has the merit of starting the appeal for £5,000 by setting forth a preliminary list of generous givers, who have promised one-tenth of the total sum asked for. It is a business document as every such appeal ought to be, and wisely expresses the hope that those friends who have helped in the past will come forward again, for the scheme of the Convalescent Home must prove of practical value to every working man who becomes an in-patient. A further point is made that, this home will prove, in reality, an extension to the Hospital work, at a cost far below what an extension of the present Hospital buildings must involve.

